# Viral Metagenomic Analysis Reveals High Prevalence of Dromedary Camel Bocavirus and Porcine Astrovirus in Bactrian Camel Intestinal Tissue

**DOI:** 10.3390/v18030302

**Published:** 2026-02-28

**Authors:** Yi Zhang, Xiaojun Ding, Xinyu Tao, Nuermaimaiti Tuohuti, Xinhao Wang, Ailixire Maimaiti, Zhanqiang Su, Xuelian Ma

**Affiliations:** 1 College of Veterinary Medicine, Xinjiang Agricultural University, Urumqi 830052, China; 120150043@xjau.edu.cn (Y.Z.); 13856580646@163.com (X.D.); 18841658067@163.com (X.T.); 320242878@xjau.edu.cn (N.T.); m13889823869@163.com (X.W.); 2 Xinjiang Key Laboratory of New Drug Research and Development for Herbivores, Urumqi 830052, China; 3 Veterinary Research Institute, Xinjiang Academy of Animal Husbandry, Urumqi 830013, China; 13629926418@163.com

**Keywords:** Bactrian camel, viral metagenomics, molecular epidemiology, dromedary camel bocavirus, porcine astrovirus, genetic evolution

## Abstract

Bactrian camels (*Camelus bactrianus*) are economically vital livestock in arid regions; however, their intestinal virome is poorly understood. We employed viral metagenomics to analyze intestinal tissue samples from deceased camels at a breeding facility in Urumqi, Xinjiang, China, and uncovered a diverse viral population dominated by dromedary camel bocavirus (DBoV1) and porcine astrovirus (PoAstV5). A molecular epidemiological survey of 261 anal swab samples collected across Xinjiang revealed prevalence rates of 36.40% (95/261) for DBoV1 and 26.44% (69/261) for PoAstV5, indicating their widespread circulation. Phylogenetic analyses of the DBoV1 NS1 and PoAstV5 ORF1a genes showed close relationships with known strains, with no evidence of recombination. This study expands the known viral spectrum of Bactrian camels, marking the first report of PoAstV5 in this species, a finding suggestive of cross-species transmission. These results enhance our understanding of camel viral diversity and provide critical data for managing enteric diseases in camel populations, with potential implications for livestock health and surveillance of zoonotic risks.

## 1. Introduction

*C. bactrianus*, members of the Camelidae family, are ancient mammals with a fossil record dating back approximately 55 million years [1]. Originating in North America, they migrated to Central Asia and were domesticated in regions such as Mongolia, Kazakhstan, and Xinjiang Province in China [1]. In Xinjiang, Bactrian camels are integral to the local economy and are valued for their milk, hair, meat, and role in transportation and tourism [2]. The growth of large-scale camel breeding, driven by animal husbandry and tourism, has heightened the need to understand and manage the diseases affecting these animals. Among these, viral infections represent a significant yet understudied threat to camel health and the sustainability of the industry [3].

Camels are recognized as reservoirs for a wide array of viruses, some of which have zoonotic potential. Notable examples include hepatitis E virus [4], herpesviruses [5], and Middle East respiratory syndrome coronavirus (MERS-CoV) [6], the latter of which has spurred extensive research into camel respiratory viromes owing to its public health implications. In contrast, the intestinal virome of camels remains poorly characterized, despite evidence of enteric diseases affecting camel populations. Recent studies have detected viruses such as the TAMdy virus and novel bunyaviruses in camels, hinting at a broader viral diversity that warrants systematic investigation [7]. Enteric viruses, in particular, may contribute to diarrhea and other gastrointestinal disorders, which are common in camel herds and can lead to significant economic loss.

Traditional viral detection methods, such as polymerase chain reaction (PCR) [8], enzyme-linked immunosorbent assays (ELISAs) [9], and cell culture [10], rely on prior knowledge of viral sequences, limiting their ability to identify novel or divergent pathogens. Viral metagenomics, which leverages high-throughput sequencing, offers a powerful alternative by enabling the unbiased detection of both known and unknown viruses [11]. This approach has revolutionized virome studies across species, from humans to livestock and wildlife, revealing unexpected viral diversity and transmission patterns [12]. Given the economic importance of Bactrian camels and the paucity of data on their intestinal viruses, we employed viral metagenomics to characterize the virome of intestinal tissue samples from deceased camels in Xinjiang, China. This study aimed to identify prevalent viral agents, assess their epidemiological distribution, and provide a foundation for mitigating viral diseases in camel populations, with potential implications for animal health and zoonotic risk assessment.

## 2. Materials and Methods

### 2.1. Sample Collection

In 2023, researchers collected 261 anal swab samples from Bactrian camels in different regions of Xinjiang, China, in different seasons (spring, summer, autumn, winter) and different breeds. Among them, 69 samples were collected from southern Xinjiang and 192 samples were collected from northern Xinjiang. By sampling season, 110 samples were collected in spring and summer, and 151 samples were collected in autumn and winter. The size of the camel herd in the sampling area is about 400 to 600 heads, and the main objects are juvenile camels (3–12 months old). Collected sick camels present with yellow watery diarrhea with normal or slightly reduced water and feed intake. Sampling was performed using sterile cotton swabs, and samples were stored at −80 °C and transported to the laboratory on dry ice. In addition, in August 2023, researchers obtained a 2–3 cm duodenal tissue sample after sterile dissection of a deceased Bactrian camel at the SL1 large-scale camel farm in Urumqi, Xinjiang, China. See Table 1 for details of the sample.

### 2.2. Histopathological Analysis

Take a 2–3 cm duodenal tissue sample, fix it in 10% neutral buffered formalin, dehydrate it through a graded ethanol series, and clear it with xylene embedded in paraffin, sectioned to a thickness of 5 μm, and stained with hematoxylin and eosin (H&E). The slides were examined under a light microscope (40× magnification) for histopathological changes.

### 2.3. Nucleic Acid Extraction, Library Preparation, and Sequencing

Samples were subjected to three freeze–thaw cycles (−80 °C to room temperature) to release the viral particles. Genomic DNA was extracted using the TIANamp Genomic DNA Kit (Tiangen Biotech, Beijing, China) according to the manufacturer’s instructions. Total RNA was extracted using the TRIzol method (China White Shark Biotechnology Co., Ltd., Bengbu, China) and reverse-transcribed to cDNA using SuperScript III (Thermo Fisher Scientific China Co., Ltd., Wuhan, China) with random hexamers. DNA and RNA were fragmented to <500 bp using a Covaris M220 (China Bailaiyuan Biotechnology Co., Ltd., Tianjin, China) with Mg^2+^. Libraries were constructed using the NEBNext^®^ Ultra™ DNA Library Prep Kit (China Jinpan Biotechnology Co., Ltd., Shanghai, China), and fragments of 200–500 bp were selected using magnetic beads. Sequencing was performed on a NovaSeq 6000 platform (China Yinmei Scientific Equipment Co., Ltd., Shanghai, China) in paired-end 150 bp mode (PE150). The RNA sequencing workflow first uses random hexamers for reverse transcription, followed by DNase treatment, then second-strand synthesis, and finally library preparation with the Nextera XT kit (Illumina, San Diego, CA, USA), with sequencing performed on the NovaSeq 6000 sequencer (Illumina).This study’s sequencing was completed at Shanghai Tanpusen Biotechnology Co., Ltd., Shanghai, China.

### 2.4. Sequence Data Quality Control

The raw sequencing reads were processed by trimming adapters and low-quality bases (30 bp, Q < 20) and filtering reads containing >10% ambiguous bases (N) using BBMap software v38.51 (https://sourceforge.net/projects/bbmap/ Accessed on 12 June 2024) [13]. Host and bacterial sequences were removed by aligning against the camel and bacterial reference genomes. Fastp v0.20.0 software (https://github.com/OpenGene/fastp/ Accessed on 12 June 2024) was used for quality control [14]. Clean reads were taxonomically classified using Kraken2 (https://github.com/DerrickWood/kraken2/ Accessed on 16 June 2024) [15], and species with a relative abundance >1% were selected as candidates to reduce false positives caused by sequence similarity.

### 2.5. Virus Validation and Molecular Epidemiology

To validate the metagenomic sequencing results, primers targeting the conserved regions of DBoV1 and PoAstV5 were designed using Primer5 (NCBI, NIH.gov) based on the intestinal tissue sequences and synthesized by Sangon Biotech (Shanghai, China). DNA and cDNA were amplified via PCR and RT-PCR, respectively, in a 15 μL reaction: 7.5 μL 2× Taq PCR Master Mix (China Tiangen Biochemical Technology Co., Ltd., Beijing, China), 4.5 μL ddH_2_O, 0.5 μL of each primer (10 μmol·L^−1^), and 2.0 μL template. The amplification conditions were as follows: 95 °C for 5 min, 35 cycles of 95 °C for 30 s, 60 °C for 30 s, and 72 °C for 1 min, followed by 72 °C for 5 min. The products were cloned into the pMD19-T vector (China Baori Medical Biotechnology Co., Ltd., Beijing, China), sequenced by Sangon Biotech (China Shenggong Bioengineering Co., Ltd., Shanghai, China), and analyzed. The primer details are listed in Table 2.

### 2.6. Phylogenetic, Recombination, and Sequence Deposition Analysis

Viral sequences identified from metagenomic data were extracted for downstream analysis. Using Clustal W settings in MEGA (v7.0), the DBoV1 NS1 and PoAstV5 ORF1a genes were aligned with reference sequences from the GenBank database (Table 3 and Table 4). Phylogenetic trees were constructed using the maximum likelihood method in MEGA software [16], and node reliability was assessed with 1000 bootstrap replicates. Nucleotide similarity was calculated using MegAlign (v7.1). Recombination events were evaluated using RDP4 (v4.101) and SimPlot (v3.5.1), comparing sequences from this study with reference strains from different hosts and geographical origins. Reference sequences and their metadata are detailed in Table 3 and Table 4. The complete sequences of DBoV1 (NS1) and PoAstV5 (ORF1a) from this study have been submitted to the database under accession numbers DBoV1 PQ588415.1 and PoAstV5 PQ615938.1, respectively.

### 2.7. Data Statistical Analysis and Charting

Epidemiological data were analyzed using IBM SPSS Statistics 27 software, and the correlation of infections across different regions, seasons, and viruses was analyzed using the Pearson chi-square test. *p* < 0.001 indicates a highly significant difference, *p* < 0.01 indicates a relatively significant difference, *p* < 0.05 indicates a significant difference, and *p* > 0.05 indicates no significant difference. Graphs and charts were created using GraphPad Prism 10.1.2 software, with significance levels marked. Heatmaps were generated using TBtools V2.1.1.

## 3. Results

### 3.1. Histopathological Analysis of Intestinal Tissue

**Figure 1:**
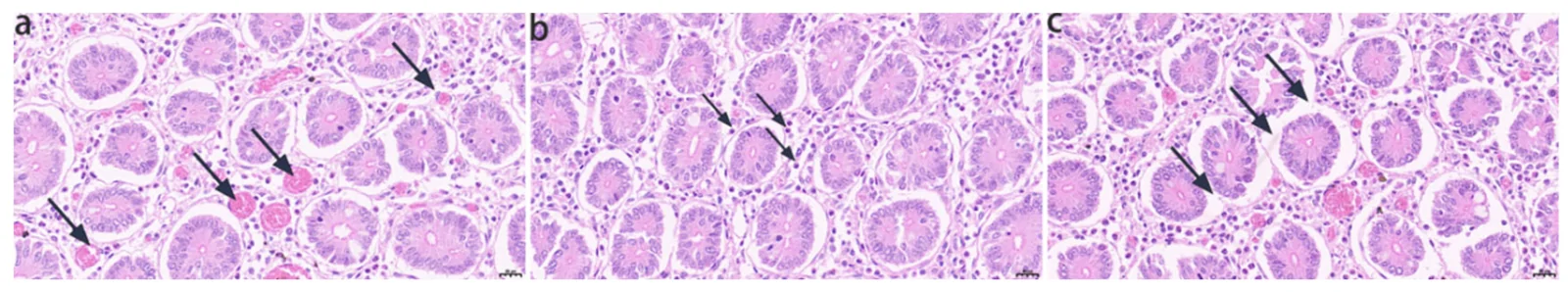
Histopathological sections of intestinal tissues from diseased Bactrian camels. (**a**) H&E-stained section showing hemorrhagic foci (arrows); (**b**) lymphocyte and plasma cell infiltration in the lamina propria (arrows); (**c**) loss of intestinal villi and disrupted architecture (arrows). Scale bar = 50 μm; 40× magnification.

*C. bactrianus* sampled for intestinal tissue exhibited clinical signs of diarrhea, including lethargy, reduced appetite, and watery or mucopurulent stools with blood. After autopsy on one camel that died in the SL1 large-scale camel farm in Urumqi, Xinjiang, histopathological examination was performed on the diseased intestinal tissue. Hematoxylin and eosin (H&E)-stained sections (5 μm, 40× magnification) revealed distinct pathological changes in the intestine. As shown in Figure 1a, the normal intestinal architecture was disrupted, with numerous hemorrhagic foci scattered throughout the tissue. Figure 1b indicates marked lymphocyte infiltration accompanied by plasma cell accumulation in the lamina propria. Figure 1c shows the loss of intestinal villi and further degradation of the tissue structure. These findings suggest severe enteritis potentially linked to viral infection.

### 3.2. Overview of the Intestinal Virome via Metagenomic Sequencing

**Figure 2:**
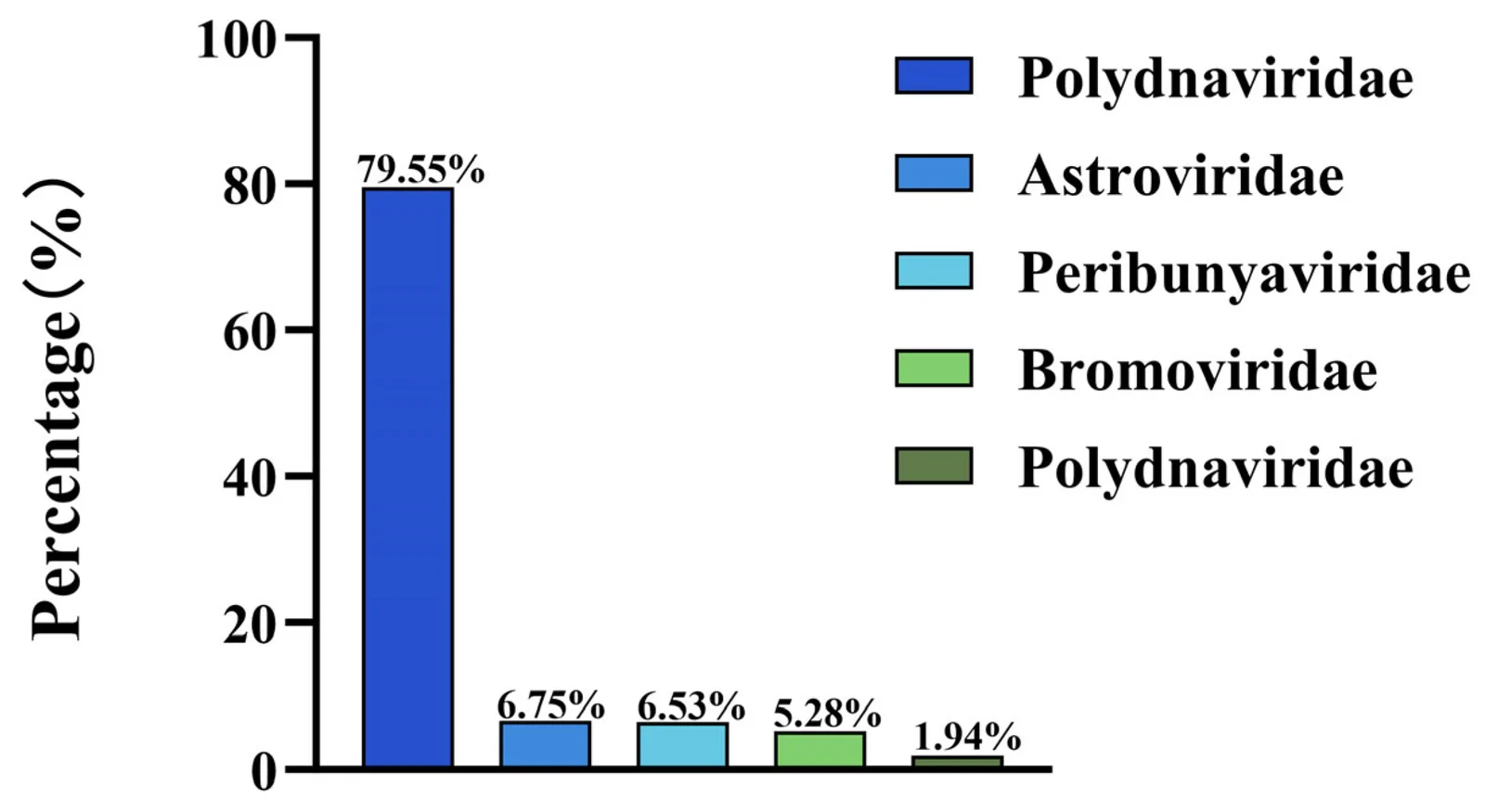
Taxonomic annotation of DNA and RNA viral families in the Bactrian camel intestinal virome. The relative abundance of the top five mammalian viral families is shown based on contig classification.

Metagenomic sequencing using the NovaSeq platform yielded 87,381 contigs, of which 1470 (≥1000 bp) were identified as viral sequences. These intestinal virome sequences were classified into three primary categories: plant-, animal-, and insect-associated. Mammalian viruses predominated, accounting for 98.06% of the viral contigs, with the remaining 1.94% attributed to non-mammalian sources. Among mammalian viruses, the top five viral families by relative abundance were Parvoviridae (79.55%), Astroviridae (6.70%), Bunyaviridae (6.53%), Bromoviridae (5.28%), and Picornaviridae (1.94%) (Figure 2). Within the family Parvoviridae, DBoV1 is the most abundant, while in the family Astroviridae, PoAstV5 stands out as the most prevalent. These findings highlight the prevalence of DNA and RNA viruses in the intestinal virome of Bactrian camels.

### 3.3. Sequence Quality Control and Decontamination

Post-sequencing quality control ensured high data integrity for both DNA and RNA viruses. For DNA sequences, the Q20 score (base call accuracy ≥ 99%) reached 97.90% after filtering, exceeding the 90% threshold for reliable data. Following the removal of ribosomal RNA, host, and bacterial sequences, 661,378 clean DNA reads remained. For RNA sequences, the Q20 score was 97.70% post-filtering, also surpassing 90%, with 700,439 clean reads retained after decontamination. The detailed quality metrics, including read counts before and after processing, ribosomal RNA removal efficiency, and GC content, are presented in Table 5. These results confirm the robustness of the sequencing dataset for downstream analyses of the virome.

### 3.4. Molecular Epidemiological Analysis of DBoV1 and PoAstV5 Prevalence

**Figure 3:**
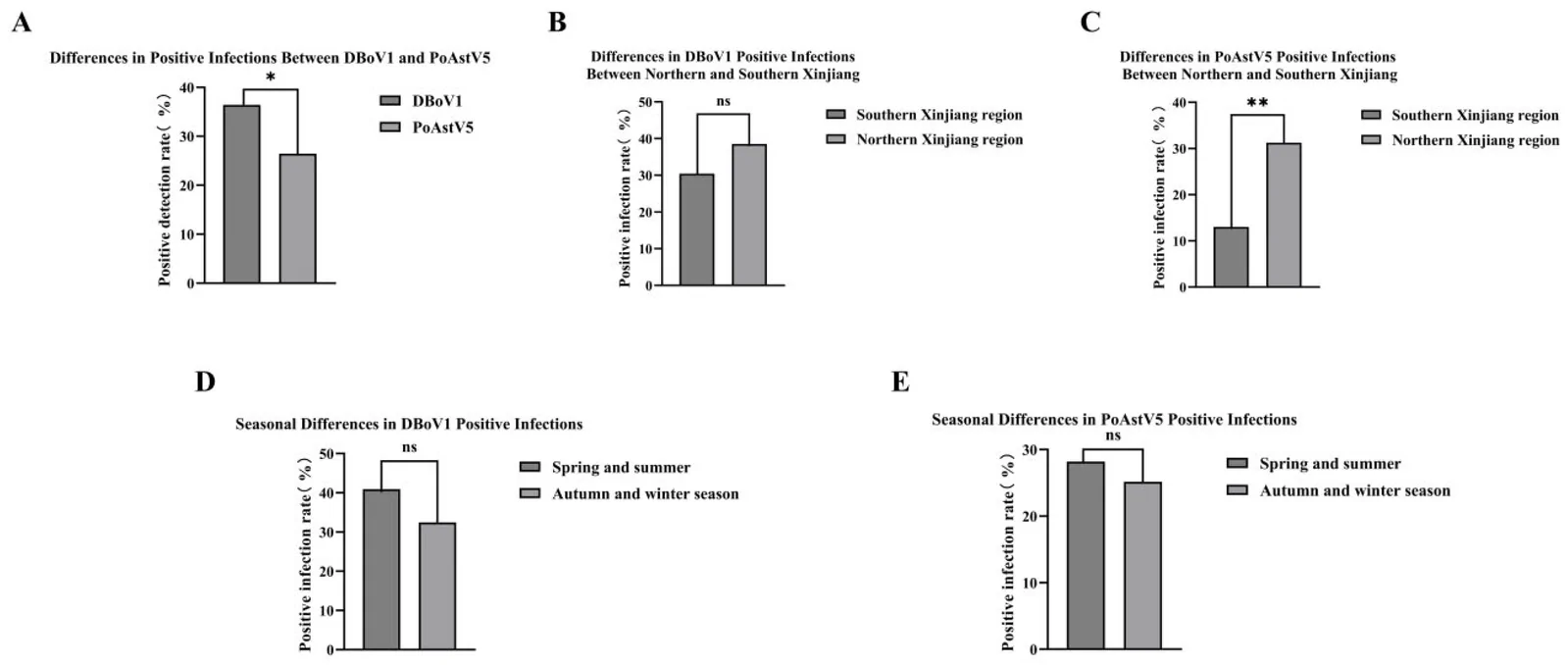
Regional and seasonal prevalence of DBoV1 and PoAstV5 in Bactrian camel anal swab samples. (**A**) Difference in positive infection rates between DBoV1 and PoAstV5, (**B**) Differences in DBoV1 Positive Infections, (**C**) Differences in PoAstV5 Positive Infections Between Northern and Southern Xinjiang, (**D**) Seasonal Differences in DBoV1 Positive Infections, (**E**) Seasonal Differences in PoAstV5 Positive Infections. Ns indicates no significant difference, * indicates a significant difference, ** indicates a moderately significant difference.

A molecular epidemiological survey was conducted to assess the prevalence of dromedary camel bocavirus (DBoV1) and porcine astrovirus (PoAstV5) in Bactrian camels (*Camelus bactrianus*) in Xinjiang, China. A total of 261 anal swab samples were collected from large-scale camel farms in 2023. PCR and RT-PCR analysis results showed that the infection rate of DBoV1 was 36%, higher than that of PoAstV5 at 26%, and the difference between the two was statistically significant (*p* < 0.05) (Figure 3A). Regional analysis of DBoV1 showed no significant difference between northern Xinjiang at 36% and southern Xinjiang at 30% (*p* > 0.05). In contrast, regional analysis of PoAstV5 showed that the infection rate in northern Xinjiang was 31%, significantly higher than in southern Xinjiang at 13% (*p* < 0.01) (Figure 3B,C), which may be related to factors such as camel population density or breeding methods. Seasonal analysis indicated that the infection rates of DBoV1 in spring and summer were 40% versus 32% in autumn and winter, and for PoAstV5 were 28% in spring and summer versus 25% in autumn and winter, with no significant differences between seasons (*p* > 0.05) (Figure 3D,E). In summary, this study confirmed the widespread transmission of DBoV1 and PoAstV5 in camel populations in Xinjiang. Overall, the prevalence of DBoV1 was higher than that of PoAstV5, and PoAstV5 showed significant regional differences in infection rate. No significant seasonal variation in infection rates of these two viruses was observed during the study period.

### 3.5. Genetic Analysis of DBoV1 and PoAstV5 Sequences

#### 3.5.1. Phylogenetic and Recombination Analysis of DBoV1 NS1 Gene

**Figure 4:**
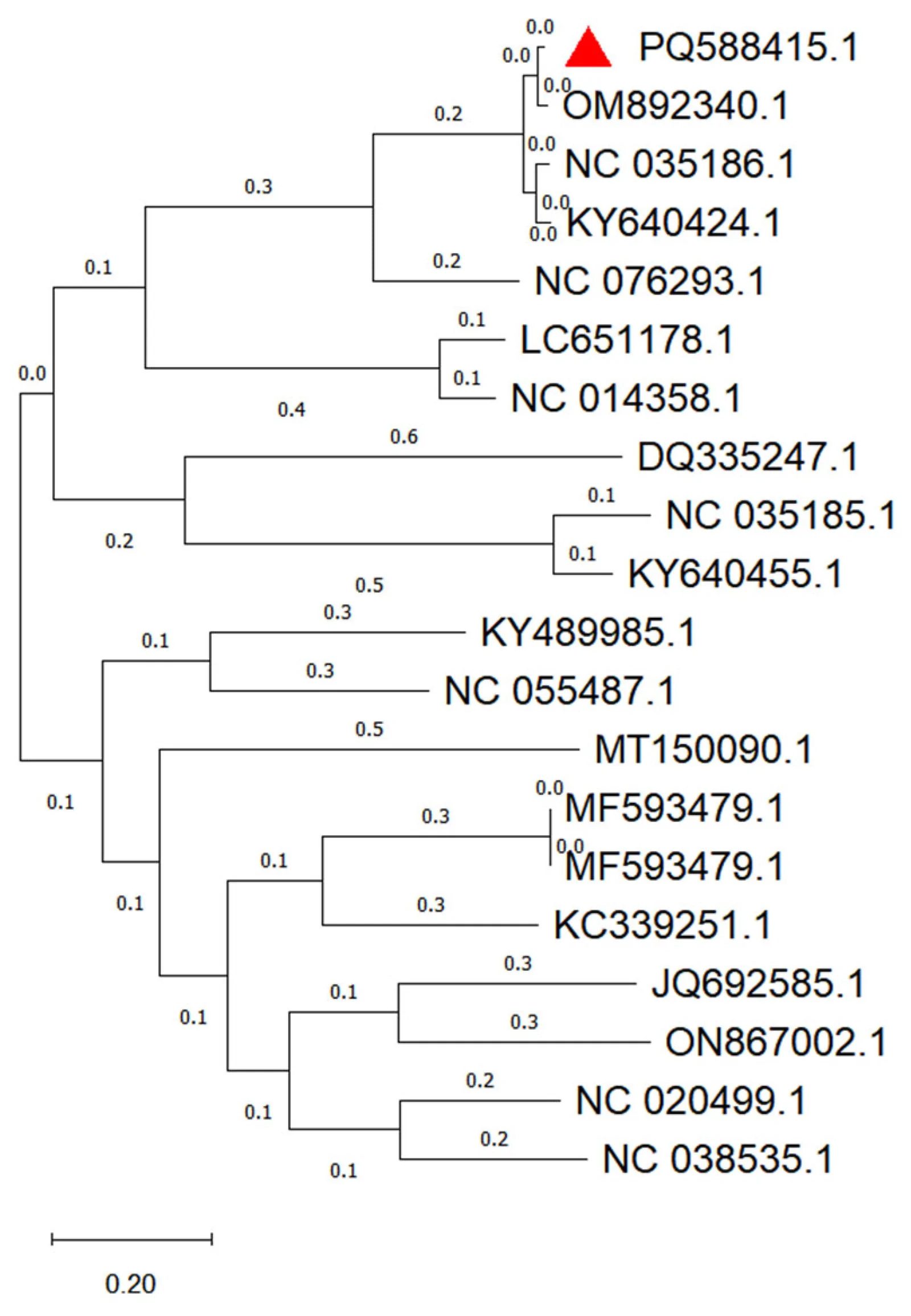
Phylogenetic tree of DBoV1 NS1 gene sequences. The red triangle represents the sequenced sequences in this study. Maximum likelihood tree showing the relationship between DBoV1/Chin/XJ-WLMQ/2023 and 19 reference strains.

Metagenomic sequencing of the DNA virus sample pool yielded two contigs of 2984 and 1180 bp. Assembly using DNAman and comparison with the NCBI database identified these as dromedary camel bocavirus (DBoV1), encompassing a complete NS1 gene and a partial NP1 gene, which was tentatively named DBoV1/Chin/XJ-WLMQ/2023. Phylogenetic analysis of the NS1 gene was performed using 19 reference strains from GenBank using MEGA (maximum likelihood, 1000 bootstrap replicates). DBoV1/Chin/XJ-WLMQ/2023 clustered most closely with a domestic camel-derived strain (OM892340.1), suggesting that it is a local variant (Figure 4). Recombination analysis using RDP4 (v4.101) and SimPlot (v3.5.1) detected no recombination events in this sequence.

#### 3.5.2. Phylogenetic and Recombination Analysis of PoAstV5 ORF1a Gene

**Figure 5:**
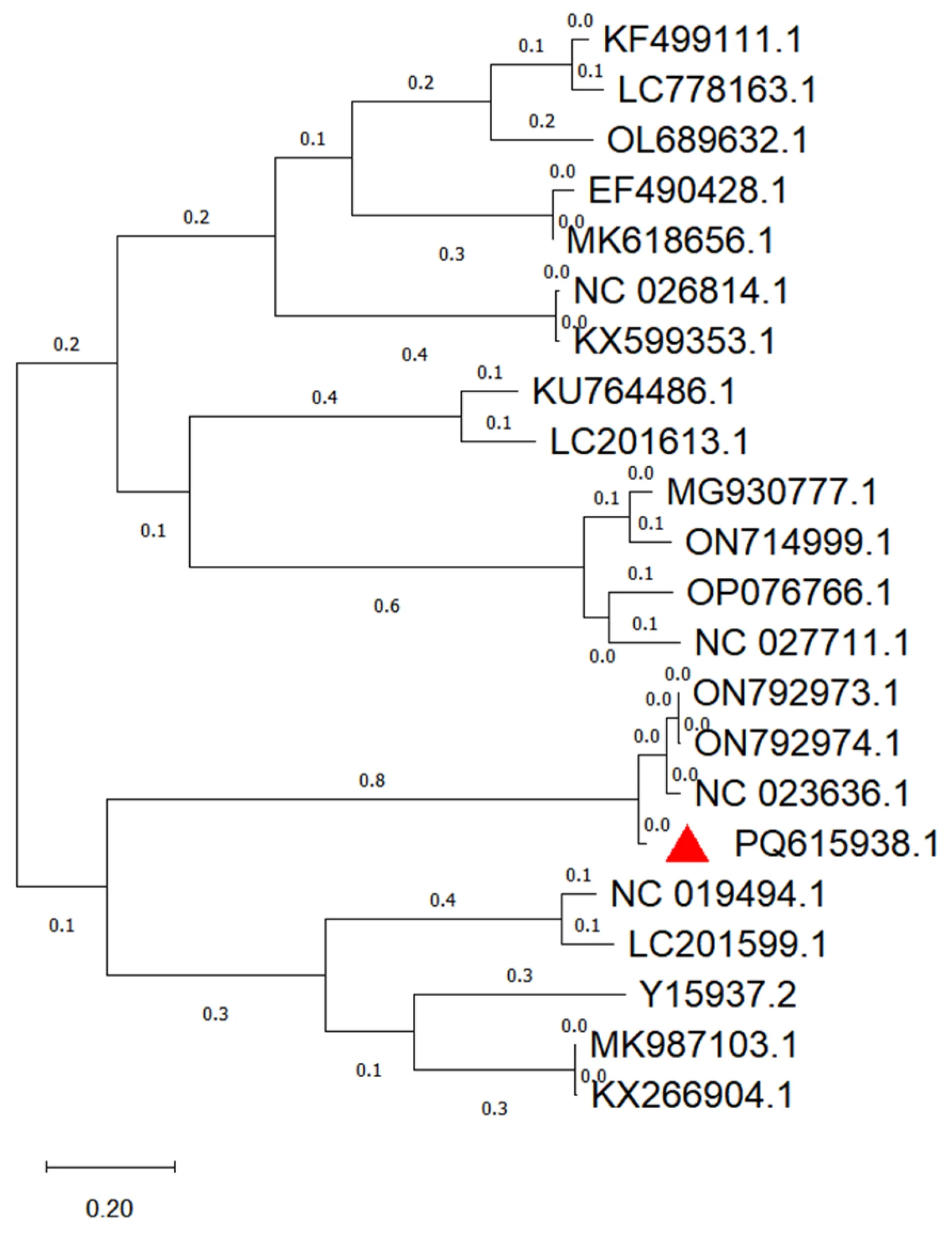
Phylogenetic tree of PoAstV5 ORF1a gene sequences. The red triangle represents the sequenced sequences in this study.Maximum likelihood tree depicting the relationship between PoAstV5/Chin/XJ-WLMQ/2023 and 21 reference strains.

From the RNA virus sample pool, a 6,401 bp contig was assembled and identified as porcine astrovirus 5 (PoAstV5) via NCBI BLAST (National Center for Biotechnology Information), containing a complete ORF1a gene, designated PoAstV5/Chin/XJ-WLMQ/2023. Phylogenetic analysis of the ORF1a gene with 21 GenBank reference strains (MEGA, maximum likelihood, 1000 bootstrap replicates) revealed the closest evolutionary relationship with a 2017 porcine strain (ON792973.1, Porcine Astrovirus 5 SP-VC9) (Figure 5). Recombination analysis using RDP4 (v4.101) and SimPlot (v3.5.1) showed no evidence of recombination in PoAstV5/Chin/XJ-WLMQ/2023.

#### 3.5.3. Homology Analysis of DBoV1 NS1 Gene

The NS1 gene of DBoV1/Chin/XJ-WLMQ/2023 was compared with GenBank sequences using MegAlign (DNASTAR Lasergene v7.1). Nucleotide homology was highest with two foreign camel-derived DBoV1 strains (KY640424.1, 94.4%; NC_035186.1, 94.2%) and a domestic parvovirus strain (OM892340.1, 97.60%). Lower homology was observed with other camel bocaviruses (NC_035185.1, 45.90%; KY640455.1, 47.10%) and a parvovirus (MF593479, 54.70%), while a canine parvovirus showed 70.90%. Homology with bocaviruses from humans, gorillas, bats, mice, and cattle ranged from 51.80% to 44.80%, indicating a significant divergence from non-camel hosts.

#### 3.5.4. Homology Analysis of PoAstV5 ORF1a Gene

The ORF1a gene of PoAstV5/Chin/XJ-WLMQ/2023 was analyzed using MegAlign (DNASTAR Lasergene v7.1) against the Nucleotide homology of three PoAstV5 strains (ON792973.1, 92.7%; ON792974.1, 92.6%; NC_023636.1, 92.3%), all porcine-derived. Homology with other astrovirus types was lower—PoAstV3 (OL689632.1, 27.1%), PoAstV2 (OL689631.1, 27.5%; ON714999.1, 27.6%), PoAstV4 (KU764486.1, 25.5%; LC201613.1, 26.0%), and unrelated astroviruses (28.4–22.9%)—confirming its classification within the PoAstV5 lineage and divergence from other genotypes.

## 4. Discussion

Viral metagenomics has emerged as a powerful tool for exploring the viral composition of animal hosts, facilitating the identification of novel and highly mutated viruses [17]. In this study, high-throughput sequencing of intestinal tissues from *C. bactrianus* in Xinjiang, China, revealed a diverse virome that included both known and previously unreported viruses. Camels are recognized hosts for multiple viral families, including Parvoviridae, Circoviridae, Poxviridae, and Hepadnaviridae. Notably, although dromedary camel bocavirus (DBoV1) was expected, the detection of porcine astrovirus 5 (PoAstV5) in camels represents a novel finding. The study area is located in a region with Islamic cultural practices, where there are no pig farms or other related farming activities in the vicinity. This environmental condition significantly reduces the possibility of direct porcine-origin contamination, providing a relatively uncomplicated background for analyzing indirect transmission pathways. Moreover, stringent decontamination procedures were implemented throughout the experiment, effectively eliminating interference from laboratory contamination. Under these premises, if PoAstV5 is still detected, the findings are more likely to be associated with other cross-species transmission routes or adaptive expansion of the viral host range. Therefore, this study aims to use this case to provide a basis for further untangling the transmission routes and evolutionary adaptability of PoAstV5 in specific environments, while highlighting its potential public health significance, which warrants in-depth investigation in the future.

Parvoviruses, among the smallest DNA animal viruses, lack an envelope, have a particle diameter of 20–26 nm, and possess a genome of approximately 5000 bp [18]. Within the Parvoviridae family, the genus *Bocaparvovirus* exhibits unique features, including an additional open reading frame (ORF3) between ORF1 and ORF2 [19]. Bocaviruses are known for their ability to transmit across species, with human bocavirus (HBoV) first identified in children’s respiratory samples in 2005 [20], followed by detection in gorillas [21], alpacas [22], cats [23], dogs [24], bats [25], pigs [26], rabbits [27], and sea lions [28]. Bocaviruses were initially documented in camels in 2014 through metagenomic analysis by Hong Kong researchers [29], with DBoV1 specifically identified in Middle Eastern dromedary camels [30]. In this study, DBoV1, the most abundant DNA virus detected (79.55% of mammalian viral contigs, Figure 2), was validated using PCR targeting its conserved NS1 gene. Molecular epidemiological surveys across Xinjiang revealed a prevalence of 36.40% (95/261), with higher rates in Northern Xinjiang (38.54%) and spring–summer seasons (40.91%) (Figure 3), contradicting the initial assumption of no regional or seasonal variation in the prevalence. Phylogenetic analysis showed that DBoV1/Chin/XJ-WLMQ/2023 was closely related to a domestic strain (OM892340.1, 93.3% homology), with no recombination events detected (Figure 4).

**Figure 6:**
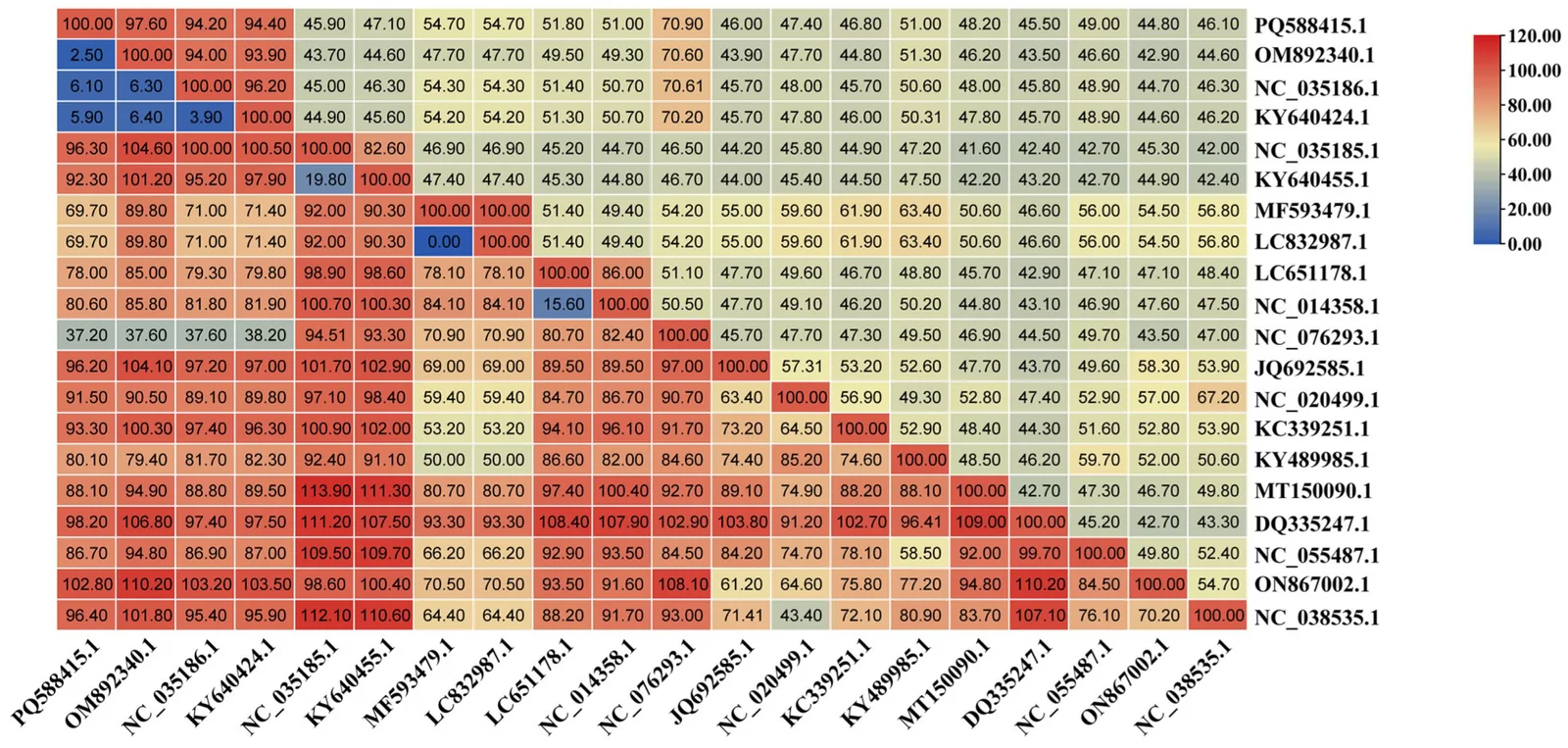
Nucleotide homology of DBoV1 NS1 with other animal bocaviruses.

**Figure 7:**
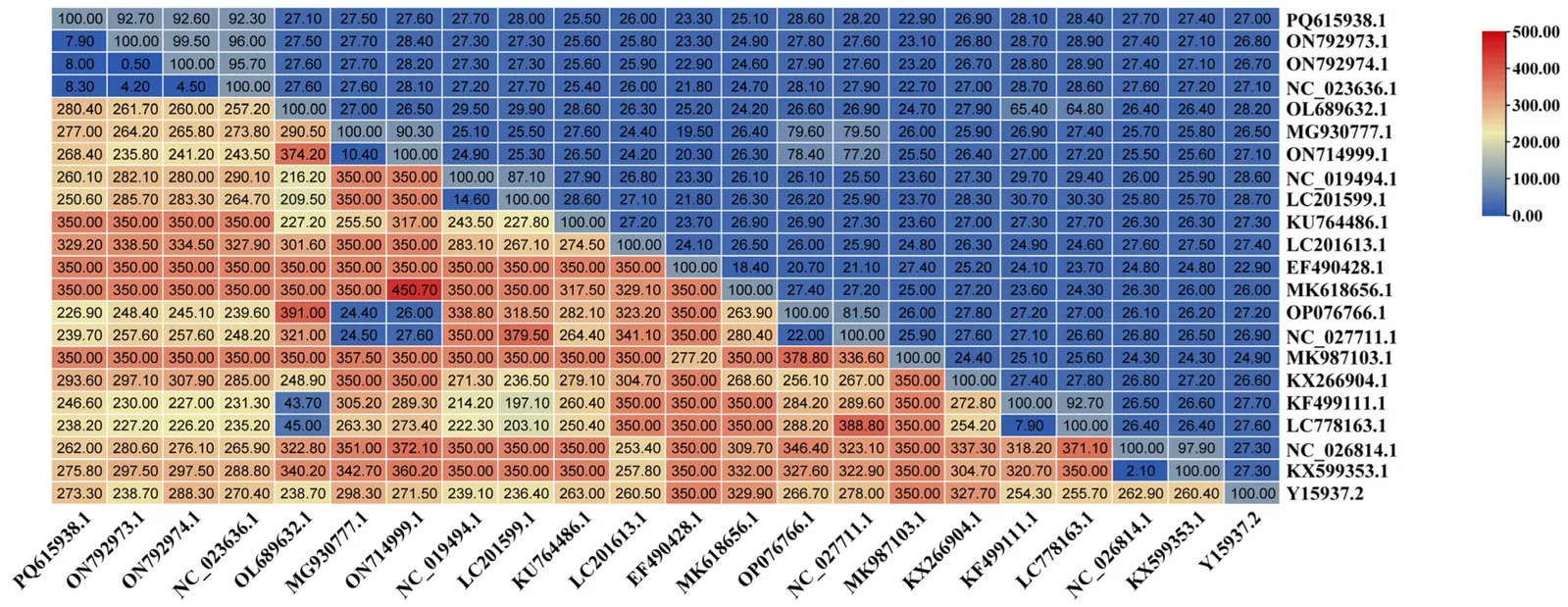
Nucleotide homology of PoAstV5 ORF1a with other animal astroviruses.

Astroviruses, small non-enveloped RNA viruses with a diameter of 28–30 nm and a genome of 6800–7900 bp [31], are classified into the *Mamastrovirus* (mammalian) and *Avastrovirus* (avian) genera [32]. Recognized as typical enteric pathogens with cross-species transmission potential, astroviruses have been reported in cats [33], dogs [34], pigs [35], sheep [36], cattle [37], and camels [38]. The identification of PoAstV5 (classified as *Mamastrovirus*) in this study was initially surprising, prompting concerns regarding sequencing errors or contamination. However, BLAST comparisons confirmed its divergence from known camel astroviruses in the NCBI database, and RT-PCR validation across 261 anal swab samples established a prevalence of 26.44% (69/261), with elevated rates in Northern Xinjiang (31.25%) and slight seasonal stability (28.18% spring–summer vs. 25.17% autumn–winter) (Figure 3). Phylogenetic analysis indicated PoAstV5/Chin/XJ-WLMQ/2023’s closest relation to a 2017 porcine strain (ON792973.1, 97.60% homology), with no recombination in ORF1a (Figure 5). Nucleotide homology analysis further revealed that DBoV1 NS1 diverged from non-camel bocaviruses (44.8–51.8%) and was moderately similar to a canine parvovirus (70.9%) (Figure 6), whereas PoAstV5 ORF1a showed high homology with porcine strains (92.3–92.7%) and low similarity to other astrovirus genotypes (22.9–28.4%) (Figure 7), supporting their respective host-specific adaptations and potential cross-species origins.

Additional viral families, such as Polyomaviridae and Bromoviridae, were detected, likely introduced via plant-associated insects in the camels’ diet, consistent with the prior findings of plant and insect viruses in the faeces of Dubai camels [29]. The unexpected 6.53% abundance of Bunyaviridae (Figure 2) without corresponding sequences suggests possible sample processing artefacts that require further validation.

This study systematically depicts the gut virome of Bactrian camels, further revealing its viral diversity and potential associations with disease. The high detection rate of DBoV1 has not been reported before, and its potential pathogenicity, especially in the complex context of camel deaths, still requires cautious evaluation. As a bocavirus, its established role in other animal intestinal diseases suggests that DBoV1 may act as a primary or co-pathogenic factor during events such as extreme climate, nutritional deficiency, or co-infection [39]. In addition, the first detection of PoAstV5 in this study, combined with its relatively high prevalence, underscores the need to further elucidate its clinical impact. Astroviruses are known intestinal pathogens; their cross-species transmission and adaptation may be associated with subclinical infections that compromise intestinal health and nutrient absorption, or, under certain conditions, cause overt gastroenteritis [40]. Such infections may weaken the host, making camels more susceptible to secondary pathogens or reducing their resistance to environmental challenges, thereby indirectly increasing the risk of death. To establish a clear pathogenic mechanism rather than merely staying at the level of correlation, future research needs to systematically reveal the direct pathogenic potential of these viruses. Specifically, the directions should include: (i) conducting longitudinal cohort studies to analyze the dynamic relationship between DBoV1 and PoAstV5 viral loads and clinical symptoms and mortality rates; (ii) developing in vivo or in vitro infection models to elucidate the patterns of viral replication and pathogenic mechanisms. In addition, the observed epidemiological patterns should be combined with farming management practices to systematically identify potential risk factors and transmission-driving conditions.

In conclusion, this study delineates the intestinal virome of Bactrian camels, enhancing our understanding of viral diversity and its association with diseases. While DBoV1’s prevalence aligns with known camel bocavirus infections, the discovery of PoAstV5 raises questions about its role in camel mortality, necessitating experimental confirmation. Metagenomic sequencing has proven instrumental in detecting these viruses, offering valuable insights into managing camel health and mitigating zoonotic risks in Xinjiang’s livestock systems [17].

## 5. Conclusions

This study utilized viral metagenomics to identify dromedary camel bocavirus (DBoV1) in *C. bactrianus* in Xinjiang, China. Additionally, genetic evolutionary analysis and molecular epidemiological surveys confirmed the presence of porcine astrovirus 5 (PoAstV5), marking its first detection in camels in this region. These findings delineate the intestinal virome of Bactrian camels and enhance our understanding of viral diversity. By establishing the prevalence and genetic profiles of DBoV1 and PoAstV5, this study provides a scientific basis for monitoring and preventing viral diseases in camel populations, laying the foundation for future studies on camel virology.

## Figures and Tables

**Table 1:** Sample Information.

Camel Anal Swab Sample			Tissue Samples
Collection Area	Harvest Season	Number	
Southern Xinjiang	spring	69	Disease intestinal tissue 1
	summertime		
	autumn		
	winter		
Northern Xinjiang	spring	192	
	summertime		
	autumn		
	winter		

**Table 2:** Primers and PCR Conditions for Validation of DBoV1 and PoAstV5.

Virus	Primer Sequences	Amplification (bp)	Annealing Temperature (°C)	Target Genes	GenBank
DBoV1	F:ACAAAGACTCGGCAGAACA	307	60	NS1	KY640425.1
	R:GAATGACCAGCACATAGACG				
PoAstV5	F:GTTCCCTTTATCCACATCC	619	60	ORF1a	ON792973.1
	R:CAAGCCTGGTAGATACGC				

**Table 3:** Reference Sequences for DBoV1 NS1 Gene Phylogenetic Analysis.

Serial Number	Strain Sequence	Login Number	Source	Year of Separation	Host
1	Dromedary camel bocaparvovirus 1	PQ588415.1	China	2024	Camel
2	Parvovirinae	OM892340.1	China	2019	Camel
3	Dromedary camel bocaparvovirus 1	NC_035186.1	Dubai	2013	Dromedary
4	Dromedary camel bocaparvovirus 2	KY640424.1	Dubai	2013	Dromedary
5	Dromedary camel bocaparvovirus 2	NC_035185.1	Dubai	2013	Dromedary
6	Dromedary camel bocaparvovirus 2	KY640455.1	Dubai	2013	Dromedary
7	Camel bocavirus 3	MF593479.1	Dubai	2015	Camel
8	Human bocavirus 1	LC832987.1	Japan	2024	Homo sapiens
9	Human bocavirus 1	LC651178.1	Japan	2020	Homo sapiens
10	Bocavirus gorilla 1	NC_014358.1	USA	2009	Gorilla gorilla
11	Vicugna pacos bocaparvovirus	NC_076293.1	USA	2018	Vicugna pacos
12	Feline bocavirus	JQ692585.1	China	2009	Felis catus
13	Canine bocavirus 1	NC_020499.1	USA	2010	Canine
14	Bat bocavirus WM40	KC339251.1	Myanmar	2008	Miniopterus fuliginosus
15	Porcine bocavirus 1	KY489985.1	Uganda	2012	Pig
16	Rabbit bocavirus	MT150090.1	China	2018	Rabbit
17	Bovine parvovirus 1	DQ335247.1	USA	2007	cattle
18	Murine bocavirus	NC_055487.1	USA	2014	Mus musculus
19	Mink bocavirus	ON867002.1	China	2021	mink
20	California sea lion bocavirus 1	NC_038535.1	USA	2010	Zalophus californianus

**Table 4:** Reference Sequences for PoAstV5 ORF1a Gene Phylogenetic Analysis.

Serial Number	Strain Sequence	Login Number	Source	Year of Separation	Host
1	Porcine astrovirus 5	PQ615938.1	China	2024	Camel
2	Porcine astrovirus 5	ON792973.1	Spain	2017	Pig
3	Porcine astrovirus 5	ON792974.1	Spain	2018	Pig
4	Porcine astrovirus 5	NC_023636.1	USA	2011	Pig
5	Porcine astrovirus 1	OL689632.1	China	2021	Pig
6	Porcine astrovirus 2	MG930777.1	Italy	2015	Pig
7	Porcine astrovirus 2	ON714999.1	Spain	2017	Pig
8	Porcine astrovirus 3	NC_019494.1	USA	2011	Pig
9	Porcine astrovirus 3	LC201599.1	Japan	2014	Pig
10	Porcine astrovirus 4	KU764486.1	USA	2015	Pig
11	Porcine astrovirus 4	LC201613.1	Japan	2015	Pig
12	Human astrovirus	EF490428.1	Madagascar	2004	Homo sapiens
13	Human astrovirus 4	MK618656.1	USA	2018	Homo sapiens
14	Bactrian camel astrovirus	OP076766.1	China	2015	Camel
15	Dromedary astrovirus	NC_027711.1	United Arab Emirates	2013	Camel
16	Bovine astrovirus	MK987103.1	Switzerland	2016	Cattle
17	Bovine astrovirus	KX266904.1	Switzerland	2015	Cattle
18	Feline astrovirus 2	KF499111.1	China	2012	Feline
19	Feline astrovirus 2	LC778163.1	Japan	2023	Feline
20	Canine astrovirus	NC_026814.1	UK	2012	Canine
21	Canine astrovirus	KX599353.1	Hungary	2012	Canine
22	Sheep astrovirus	Y15937.2	United Kingdom	2000	Sheep

**Table 5:** Quality Control and Decontamination Metrics for DNA and RNA Sequencing Data.

Project	DNA		RNA	
	Before Processing	After Processing	Before Processing	After Processing
Q20	96.90%	97.90%	96.50%	97.70%
The number of reads before and after filtering	36,557,148	35,523,736	433,499,644	42,094,836
Reads after removal of RiboSome RNA	17,672,525 (95.50%)		20,877,092 (99.19%)	
Remove the host filter the bacteria after the reading	661,378 (3.72%)		700,439 (3.33%)	
GC%	51.74%	51.55%	55.06%	54.63%

Note: Q20: The probability of error recognition is 1%—that is, the error rate is 1%, or the correct rate is 99%—and the quality of Q20 is higher than 90%; the data quality is good.

## Data Availability

Virus clones are available upon request. The sequences generated in this study are available at GenBank.
